# Health workers’ experiences of coping with the Ebola epidemic in Sierra Leone’s health system: a qualitative study

**DOI:** 10.1186/s12913-018-3072-3

**Published:** 2018-04-05

**Authors:** Joanna Raven, Haja Wurie, Sophie Witter

**Affiliations:** 1 Department of International Public Health, Liverpool School of Tropical Medicine, Liverpool, L3 5QA UK; 2 College of Medicine and Allied Health Sciences, University of Sierra Leone, New England, Freetown, Sierra Leone; 3 Institute for Global Health and Development, Queen Margaret University, Edinburgh, UK

**Keywords:** Health workforce, Resilience, Epidemic, Sierra Leone

## Abstract

**Background:**

The 2014 Ebola Virus Disease epidemic evolved in alarming ways in Sierra Leone spreading to all districts. The country struggled to control it against a backdrop of a health system that was already over-burdened. Health workers play an important role during epidemics but there is limited research on how they cope during health epidemics in fragile states. This paper explores the challenges faced by health workers and their coping strategies during the Ebola outbreak in four districts – Bonthe, Kenema, Koinadugu and Western Area - of Sierra Leone.

**Methods:**

We used a qualitative study design: key informant interviews (*n* = 19) with members of the District Health Management Teams and local councils, health facility managers and international partners; and in depth interviews with health workers (*n* = 25) working in public health facilities and international health workers involved with the treatment of Ebola patients.

**Results:**

There were several important coping strategies including those that drew upon existing mechanisms: being sustained by religion, a sense of serving their country and community, and peer and family support. Externally derived strategies included: training which built health worker confidence in providing care; provision of equipment to do their job safely; a social media platform which helped health workers deal with challenges; workshops that provided ways to deal with the stigma associated with being a health worker; and the risk allowance, which motivated staff to work in facilities and provided an additional income source.

**Conclusions:**

Supportive supervision, peer support networks and better use of communication technology should be pursued, alongside a programme for rebuilding trusting relations with community structures. The challenge is building these mechanisms into routine systems, pre-empting shocks, rather than waiting to respond belatedly to crises.

## Background

In Sierra Leone, the Ebola Virus Disease (EVD) outbreak, which started in 2014 and officially ended in 2016, evolved in alarming ways, spreading nationwide. The country struggled to control the escalating outbreak against a backdrop of a health system that was already over-burdened [1, 2]. Being a post-conflict country, the health system in Sierra Leone can be described as fragile and sub-optimal in the face of a disease outbreak as shown by its poor health outcomes. The health workforce was inadequate and ill-equipped to deal with the outbreak including limited national infectious disease expertise. In 2010, the population density of doctors was 2/100,000 population compared to the World Health Organisation’s (WHO) recommended threshold of 23/100,000 [3]. In addition, only one hospital in Sierra Leone had a functional infectious disease unit, the Kenema Government Hospital’s Lassa Fever Unit, headed by the late Dr. Sheik Umar Khan. From this disadvantaged stand-point, the national capacity to manage the outbreak was further deflated when Dr. Khan, Sierra Leone’s only specialist virologist for viral haemorrhagic fever, succumbed to the virus, very early into the outbreak.

The outbreak quickly led to considerable morbidity and mortality, exacerbated by the weak health system with inadequate numbers of health personnel, surveillance systems, diagnostic facilities, isolation wards and protective equipment. A reported total of 3956 people died in Sierra Leone during the outbreak [4], but this does not include deaths which were not reported by family members due to fear and other socio-cultural factors, for example, burying their loved ones according to religious practices, which was prohibited at that time. Health workers were 21–32 times more likely to be infected with Ebola than the general adult population [5]. An unprecedented number of health workers were infected, with an estimated 221 deaths [4], which is an estimated 21% of the overall health work force in Sierra Leone [6].

Health workers are at the centre of health systems. In Sierra Leone, efforts made in the post-conflict period to strengthen human resources for health (HRH) suffered a major knock by the EVD outbreak [7]. Recent papers have highlighted that a weak health system cannot be resilient and cope with crises such as an EVD outbreak, and called for ‘national governments, assisted by external partners, to develop and implement strategies to make their health systems stronger and more resilient’ [2, 8, 9].

Research from high income settings identifies factors that influence health worker behaviour during epidemics: fear of contagion, concern for family health, interpersonal isolation, quarantine, trust in and support from their organisation, information about risks and what is expected of them, and stigma [10–13]. Risk mitigating strategies included organisational implementation of infection prevention control (IPC) measures, avoidance of patients, and complying with personal protective equipment (PPE) [10]. They called for more research into factors that influence healthcare workers’ decisions to provide care at the frontline.

However, there is limited research on how health workers experience and cope during health epidemics in fragile states. Previous studies highlight how individuals who survive Ebola (whether patients or health workers) often face stigmatization by family, co-workers and communities, depression and difficulties in reintegration into society [14–16]. Commitment to their profession was identified as the underlying motivation to continue to work despite lack of PPE and other resources necessary to provide care safely [14]. A recent study in Sierra Leone examined how health workers in peripheral health care facilities in two districts in Sierra Leone experienced the changes in their professional and personal lives during the EVD outbreak [17]. Key findings included a weakened sense of trust within and across health facilities, providers, communities and households, and feelings of stigmatization, isolation and sadness amongst health workers. Enhanced psychosocial support for not only providers working in designated Ebola treatment and care facilities but also those working in facilities that are not specifically for Ebola management is needed.

Our earlier research in Sierra Leone, under the REBUILD programme (this DfID funded programme is a research consortium working in four post conflict countries generating evidence to rebuilding health systems post conflict and post crisis, and contributing towards health systems strengthening), investigated the post-crisis dynamics for human resources for health and ultimately how to reach and maintain incentives to support access to affordable, appropriate and equitable health services [18]. In particular, it explored health worker experiences of working during and post conflict, identified factors that motivated or demotivated them to provide services, and their coping strategies through a combination of qualitative and quantitative methods. The research highlighted that developing the capacity of health workers and developing a motivated health workforce is an ongoing issue. We build on this by exploring health workers experiences during another type of crisis – the EVD outbreak.

In this study we explore the challenges faced by healthcare staff working in government facilities, which took on the brunt of managing the EVD outbreak, and their coping strategies in four districts of Sierra Leone: Western Area, Kenema, Bonthe and Koinadugu. Understanding how the health system responded to the outbreak, from a health workers’ perspective, is important in rebuilding the health sector in the post-Ebola phase, and building resilience to such shocks in the future.

## Methods

This study was conducted between March and May 2015. It used qualitative research methods - in depth interviews (IDI) with health workers and key informant interviews (KII) – to explore their experiences before and during the EVD outbreak, that is from 2013 to March 2015. Qualitative interviews facilitate generation of in-depth and contextual information about an individual’s experience, beliefs and perceptions as well as exploration of reasons behind their answers through probing questions [19, 20].

The study was conducted in four districts in Sierra Leone. The selected study districts were the same as those selected for the ReBUILD health worker incentive project, as they represent different regions of Sierra Leone with different timing and extent of outbreak, relationships with district and facility managers made it easier to conduct the study, and it allowed us to build on existing findings. The districts were:

1. Western Area (Urban/Rural) District – high numbers of EVD patients and epicenter during the outbreak (between 501 and 4000 confirmed cases), large urban and rural populations and referral hospitals
2. Kenema District (Eastern Region) – high numbers of EVD patients and epicenter during the outbreak (between 501 and 4000 confirmed cases), large urban and rural populations and referral hospital
3. Bonthe District (Southern Region) – low numbers of EVD patients (between 1 and 5 confirmed cases), hard to reach area as riverine
4. Koinadugu District (Northern Region) – hit by Ebola in the later stages of the epidemic (between 101 and 501 confirmed cases), no treatment centre, hard to reach as mountainous and 300 km from Freetown

### In depth interviews with health workers

We conducted IDIs with frontline government health workers who provided clinical services, to explore their perceptions and experiences of the EVD outbreak in Sierra Leone and the impact of the outbreak on them, and to identify any coping mechanisms that they used. Four groups of health workers were selected:

1. *Health workers who were interviewed for the ReBUILD health worker incentive study* [13] we followed up as many as possible of the 23 participants included in the health worker incentive study. Even when not available for interview, we tried to document their current status, where possible.
2. *National health workers working in Ebola treatment or isolation centres:* we selected 2 health workers working in each centre in the most hit study districts, Western Area and Kenema.
3. *National health workers working in other health facilities:* we selected 2 health workers working in a district hospital and community health centre in each study district. This group allowed us to understand the wider effects of EVD, beyond the specific treatment centres.
4. *International health workers working in Ebola treatment or isolation centres:* we selected international health workers working in these centres in the most hit study districts, Western Area and Kenema. These interviews captured the perceptions of outsiders with operational insights on the current functioning of service delivery in the districts. As health workers who have not worked in the Sierra Leone health system, they provided a unique and important perspective on how health workers coped with responding to the outbreak, and ways to rebuild the health system post- Ebola.

### Key informant interviews

The key informants (KI) were purposefully selected based on them being a member of the District Health Management Team (DHMT) or local councils, health facility managers and international partners working in the study districts. The members of the DHMT and local councils do not have clinical roles but are involved in organising and managing the health care services including health workers. The health facility managers and the international partners play both a clinical and managerial role. They had a detailed knowledge of the health system response to the outbreak and could provide perceptions and experiences of the response.

### Data collection and analysis

The interviews, were conducted in English, in a private room in the health facility, office or in their home where the participant felt most comfortable. Separate topic guides were used for the in-depth interviews with health workers and key informant interviews. The topic guides for the in-depth interviews covered health workers’ perceptions and experiences of working during the ongoing Ebola outbreak, any constraints that they faced, challenges in the health systems, their coping mechanisms, and options to increase the resilience of workers and the health system in the future. The topic guides for the key informants included the following areas: perceptions and experiences of the Ebola outbreak; its impact on health workers; constraints, challenges and opportunities in relation to leadership and governance, health workforce and service delivery during the Ebola outbreak; and options to increase the resilience of workers and the health system in the post Ebola phase.

The interviews were digitally recorded after gaining permission from the participants. The recordings of the interviews were transcribed verbatim and analysed using the framework approach which facilitates rigorous and transparent analysis [21]. The coding framework was developed using themes emerging from the data, the topic guides and study objectives. The authors applied the coding framework to the transcripts, charts were developed for each theme, and these charts were used to describe the themes. NVIVO 10 was used to support the analysis.

### Ethics

Ethical approval was obtained from the Sierra Leone Scientific and Ethics Committee and the Liverpool School of Tropical Medicine Research Ethics Committee. Rigorous informed consent process was followed: all participants were given verbal and detailed written information about the nature and purpose of the research before taking part; participants were made aware of their right to decline to answer questions, and were assured that measures are in place to anonymise responses. All participants gave written consent. All data were anonymised.

## Results

### Details of participants

A total of 25 health workers were interviewed, comprising of 10 male and 15 female health workers (2 in Bonthe, 7 in Kenema, 6 in Koinadugu, and 10 in Western Region). Table 1 provides details of the participants. Of the 23 health workers included in the ReBUILD health worker incentive study, only 8 were available to participate in this study, due to a number of reasons. These reasons included: death, one from EVD; unwilling to participate in the study; health worker now working in a different health facility that is not in the chosen four study districts; and health workers away from their work stations at the time of the study. Only 2 health workers were interviewed in Bonthe district as there were no Ebola treatment centres or isolation centres in this district, due to the low number of EVD cases reported in this district. In addition, we were unfortunately unable to follow up the health workers who were in the ReBUILD health worker incentive study, as they were engaged at the last minute in EVD related training in another district. A total of 19 KIIs were conducted, of whom 13 were male and 6 were female (Table 1).

**Table 1:** Study participants in the 4 study districts

	Health workers included in the study (by cadre and sex)				Total	Key informant interviews (by sex)				Total	Total per district
	From the HW incentive study	National HWs from treatment or isolation centres	National HWs from other health facilities	International HWs from in treatment or isolation centres		DHMT	Local council	Health facility manager	International partner		
Bonthe^1^	0	0	2 (Nurse F; CHO M)	0	2	1 (M)	1 (M)	2 (M)	1 (F)	5	7
Kenema^2^	1 (Doctor M)	2 (Nurse M; Nurse aid F)	2 (MCH aid F; Lab technician F)	2 (Doctor M; Midwife F)	7	1 (M)	0	2 (1 M; 1F)	1 (M)	4	11
Koinadugu^3^	3 (CHO M; 2xNurse F)		3 (SECHN F; MCH Aid F; CHA M)	0	6	1 (M)	1 (F)	2 (M)	1 (M)	5	11
Western Area^4^	4 {3× Nurse (2F; 1 M) Midwife F}	3 (Nurse F; 2× Doctor, M)	2 (Midwife F; MCH aid F)	1 (Doctor M)	10	1 (F)	1 (M)	2 (1 M; 1F)	1 (F)	5	15
GRAND TOTAL					25					19	44

Key: 1 = Low numbers of EVD patients; hard to reach district; 2 = High numbers of EVD patients; epicentre during outbreak; 3 = Hit by EVD in later stages of outbreak; hard to reach district; 4 = High numbers of EVD patients; epicentre during outbreak

*CHO* Community Health officer, *MCH Aid* maternal and child health aid, *SECHN* State enrolled community health nurse, *CHA* Community Health Assistant, *M* male, *F* female

Four key areas emerged from the data: the preparedness of the health system to manage the EVD outbreak; the impact of the outbreak on health care staff; the coping strategies of staff; and ebola response interventions. Although we looked for variations between districts, gender, type of health worker and KIs, there were no strong thematic differences.

### Preparedness of the health system to manage EVD outbreak

Specific challenges related to readiness of the system to manage the EVD outbreak were reported. There was a lack of a triage system, isolation and treatment beds, IPC training, and PPEs. At the start of the EVD outbreak there was poor practice in terms of IPC, such as limited handwashing and wearing gloves.

Respondents talked of fighting a battle without equipment due to limited numbers of laboratories, instruments and supplies. They lacked enough gloves for basic protection: one health worker explained that he was allocated 2 pairs of gloves per week, which she perceived as being inadequate for the work she was doing. There were insufficient instruments or materials such as Caesarean Section kits or suction catheters, resulting in inappropriate reuse or inadequate sterilisation, putting both patients and staff at risk of contracting Ebola, and other diseases.

*You need to have the necessary equipment to fight. What has been the problem is that even when there is this readiness of facing this battle we have not been given the proper equipment to fight. (KI, Bonthe)*

A lack of knowledge and misconceptions about Ebola amongst health workers also contributed to fear of the disease and uncertainty on how to protect themselves from infection as well as care for patients. This slowly improved over time, as health workers learned more about Ebola and how to protect themselves.

*Lack of knowledge was what was prompting the fear and that continued because of all sorts of messages. Everybody was coming with their own ideas - it was not curable, this is what will happen, you should not do it that way… but when we started learning about the Ebola it became better. (HW, Western Area)*

### Impact of the outbreak on health workers

Respondents reported several negative effects on health workers.

#### Breakdown of trust

A breakdown of trust was reported between neighbours /communities and health workers. Many community members believed that Ebola was spread by health workers through contact, exchanging blood or injections, and were frightened of health workers dressed in protective gear. For many health workers, this resulted in a sense of isolation and in some cases being ostracised for example, not being allowed to use the village well for their water, being asked to leave their rented accommodation, and not being allowed to use taxis.

In addition, many health workers reported that they were also afraid of patients. This was particularly the case at the start of the outbreak, when health workers were ill equipped in terms of knowledge and supplies to protect themselves from infection. A few health workers also reported that patients did not always answer truthfully about their symptoms during assessment, and this exacerbated their lack of trust in the community.

*It really affected my profession … I cannot wear my uniform to work. On paper you have to start asking questions - have you been down with a fever, has somebody died close to you. Some people became very suspicious of us and so they did not want us. So that affected me a lot. I love my patients to have confidence in me, that one was broken. (HW, Western Area)*

*… colleagues in the general ward they were really intimidating us. If I walked through this corridor, they will just move and just give a space for me to pass. (HW, Kenema)*

#### Isolation from families

Ebola affected how health workers interacted with their families. Health workers kept away from their families until they had changed their clothing and washed thoroughly. They were reluctant to have close contact and play with their children. Others spoke of not visiting home for long periods.

Families of health workers were very worried about their relatives going to work in the facilities, either Ebola treatment and holding centres or “normal” health facilities. They were worried that the health workers would contract Ebola and either die or transmit it to other relatives.

*I left home on August 7th 2014 and since then I’ve not been back because I didn’t want to work with patients and go home and if I should fall sick, if its Ebola then my family will have to be in quarantine for 21 days, which would mean my sisters would not go to work, my brothers would not go to work, my mother would not go to work. (HW, Western Area)*

Some health workers reported that they were pressurised by their family to discontinue working but they continued to do their job as they felt that it was their duty. Some respondents reported that other health workers did give up work and stayed at home.

*My family, my friends they told me don’t go to the centre stay […]This is Ebola time and this Ebola is so serious. Most likely the place where you are going […] they have suspected cases there and you are going, don’t go yet. Your life is more important. I said no I’m going. (HW, Koinadugu)*

#### Fear of being infected

Health workers reported great fear of contracting Ebola. They were worried about how well they followed infection control practices. Many reported constantly looking for Ebola symptoms.

*So all the time, round the clock you have to be alert. Knowing the signs and symptoms of Ebola, when you go home, dust during the day affected your eyes and you started blinking. You sit at home, maybe this is Ebola, you begin to count 21 days. (HW, Kenema)*

#### Trauma from watching colleagues die

Health workers saw many colleagues, as well as relatives and community members die. They spoke of caring for colleagues as they died and only later realising that they had Ebola.

*“We lost our colleague here and I was the person that stayed with that colleague for the rest of the day. When I went home they called me, they text me that she’s gone, she’s dead… three days or four days after, the result was out …saying she was positive, Ebola positive. I started thinking about myself … the time that I was taking care of Nurse xxx, did I dress properly, how did I dress. So I was confused, my mind was scattered. After 2 days I became sick, the mind was sick, everything about me was sick.” (HW, Western Area)*

#### Economic hardship

Economic hardship due to reduced earnings was common: the no touch policy, introduced and implemented during the EVD outbreak to deter unprotected bodily contact, and the general lack of mistrust between health workers and service users meant health workers could no longer engage in a second health related incoming generating activity, a practice that is common in Sierra Leone. Delays in receiving the risk allowance also contributed to their economic hardship (see Risk allowance section for more details).

*Many health workers, their basic earning power decreased as a result of Ebola. So it has this economic impact which has an attendant problem on the family livelihoods. (KI, Western Area)*

#### Increased stress and workload

In the workplace, health workers often reported increased stress and workload, and a continued struggle to get the supplies they needed. Some reported distrust between staff – for example, staff from the general wards avoided staff from the treatment centres as they were frightened that they would transmit the virus to them. Managers were supportive in some cases where they talked to them regularly and encouraged them to keep working. However, in other settings, health workers reported that managers gave instructions, but rarely came to give encouragement.

### Ebola response interventions

#### Risk allowance

Health workers were asked about how they coped financially during the outbreak. They explained that the costs of living such as food and transport had increased dramatically during the outbreak. Many health workers reported difficulty in coping with increased living costs and rationed their food and controlled their movements.

A risk allowance was provided to supplement health worker salary. The risk allowance rates ranged from 500,000 Leones (approximately $70) per week for doctors, nurses, midwives, community health officers working in treatment centres and community care centres and all members of the burial team, to 100,000 Leones (approximately $13) for contact tracers. The monthly salary for a Grade 7 nurse is 1,814,400 Leones (approximately $240), and for a grade 1 level staff such as a cleaner is 480,000 Leones (approximately $64). The risk allowance motivated some staff to work in the facilities and provided an additional income source which helped them cope to some extent with the increased cost of living. However, there were also concerns about delays and gaps in provision, as well as who received the allowance and how it was set.

*If a junior like a cleaner is having 800 thousand Leones [equivalent to $100] on a monthly basis, you the senior staff also have 800 thousand on a monthly basis that is not commensurate to your work, there should be some difference at least. (KI, Koinadugu)*

#### Training and workshops

Training assisted health workers overcome fear and become more confident about providing care. Training was particularly effective when it was coupled with the supply of essential resources such as PPEs, bleach and gloves. For those working in hard to reach areas, the managers of facilities passed on any training to the rest of the team.

As the outbreak continued, health workers reported that their skills and knowledge in triage, management of Ebola, and IPC measures improved over time through training and clinical practice. Some reported that they would be better equipped to manage outbreaks in the future.

*You know the positive aspect is that I have gained a lot of confidence, experience, I have a vast experience you know and I know some day if there’s any outbreak like this I will be able save lives. (HW, Western Area)*

Psychosocial support was also given to health workers in workshops. These workshops were conducted during the middle and later stages of the outbreak. Social workers and mental health workers helped them cope with the stigma of being a health worker during an epidemic.

*We have social workers and mental health workers who conduct training, talk to us about the stigmatisation, what not to do, what to do, so really we are now calm. (KI, Bonthe)*

### Coping strategies of staff

Several coping strategies were reported by health workers during the outbreak.

#### Sense of duty to serve their country and their communities

Many health workers identified the EVD outbreak as a national crisis. They reported a sense of duty to serve their country and their communities during this crisis*.* They felt that they should continue to provide care despite the many challenges including dangers to their own health.

*We just feel that we are Sierra Leoneans and we should, if we do not go into and help our people who will do that. (HW, Bonthe)*

*We are working because we work in the interests of the people as it affects the community greatly. Affect me, my people, community greatly. (HW, Koinadugu).*

#### Peer and family support

Health workers spoke of the importance of peer support. Health workers encouraged each other, watched how they were managing patients and reminded them about IPC. They also reported receiving support from senior health workers and managers: they were hesitant in treating patients on their own but became more confident after these patients were seen by a more senior health worker.

*Like in my hospital I was really impressed with my doctor and my matron. They came in at the time we needed them most. Most of the times when these patients have come around, we feel frightened to go there but if they first get there we feel that we are safe. (HW, Bonthe)*

Some health workers spoke of their family support, and how their words of encouragement helped them cope with their work including loss of colleagues.

*Yes our friends come, my family, my family called to support us to make sure we take care and then give us words of encouragement you know, whenever we lost our colleagues. (HW, Kenema 3)*

#### Social media platform

A social media platform was set up by some frontline health workers during the outbreak to help them cope with the stresses and challenges of working during the Ebola outbreak.

*We have a WhatsApp group about Ebola fighters and you should see the text messages. It’s incredible like you get up in the morning at 6 and all of them, it’s like ‘please remember to play safe in the unit’,’ we have to take care of each other’, ‘come on guys we can do it we are going to kick Ebola out of the country’. (KI, Western Area)*

#### Religion

Many health workers reported that their religious belief helped them cope with seeing patients and colleagues dying from Ebola. Health workers often prayed together before starting work.

*My confidence is with God because really it is not easy. Colleagues are dying, other people are dying but I said now if I left who can save the lives. So I will just believe in God to do my job. (HW, Kenema)*

## Discussion

This study engaged with health managers and staff working in routine and Ebola treatment centres in Sierra Leone, and documented their views and experiences not just on the epidemic but also how they coped through it, and what they require in the health sector reconstruction phase. There were challenges related to the readiness of the system to manage the Ebola outbreak, as well as effects on the personal and professional lives of health workers. Despite these challenges, huge resilience was evident – resilience being understood here as the ability to absorb shocks and maintain services in the face of them [2] - facilitated through training, workshops, social media platform, support from colleagues, families and communities, religion, and the risk allowance. These findings resonate with wider literature about how health staff cope with different types of shocks [22].

In terms of readiness, the lack of triage facilities, of training in IPC, of PPEs and other enablers, is consistent with other reports on the Ebola epidemic [8, 23, 24]. A survey carried out in all 1185 Primary Health Units in October 2014 found that health personnel in 37% of the Primary Health Units felt they were not provided with adequate training on Ebola, 15% identified lack of information about Ebola as a challenge, an overwhelming 90% felt fear/misconception as being the main challenge confronted by the health system to fight Ebola, 87% reported lack of protective gear as a large gap and 26% reported lack of medicines as a big constraint [25]. These findings resonate with McMahon et al. [17] but also with other studies exploring health workers’ experiences in severe respiratory epidemics [10–13]. The response to the epidemic will also have been affected by the underlying conditions and incentives facing health workers in Sierra Leone (e.g. lack of training and career opportunities, difficult working conditions, long working hours and limited financial and other rewards), even before the epidemic [18], which were more challenging in rural areas where the epidemic emerged.

This study provides new insights into how health workers adopted coping strategies in Ebola epidemic in Sierra Leone. Over time, the health workers were able to cope better with the outbreak. Health workers reported that at the start of the outbreak, there was a lot of fear amongst health workers about Ebola. Training and workshops, as well as increased clinical experience, improved their knowledge and skills, which relieved this fear and helped them better cope with the outbreak. In addition, peer support and psychosocial support workshops helped health workers cope with the stigma of being health workers during an epidemic. Externally derived coping strategies included training, workshops, financial support, and the social media platform; and those strategies that draw upon existing mechanisms such as being sustained by religion, a sense of serving their country, peer support and family support. These are similar to the coping strategies documented in an earlier study of coping with conflict in Uganda [26] and in Sierra Leone [18], with the addition of innovations made during the Ebola outbreak (e.g. the social media platform, the risk allowance). Peer and manager support came out strongly from the interviews. In the context of an emergency, it is possible that non-financial, professional support approaches are more powerful motivators than in stable contexts.

Several recommendations for rebuilding a resilient health system post-EVD outbreak emerge from this study, including maintaining and building on IPC practices in order to contain future outbreaks through in-service training and supportive supervision, maintaining isolation wards with essential equipment, and institutionalising the triage system in all facilities. Some of the infrastructure which was created in response to Ebola should now be effectively incorporated into the health system, and the outstanding gap areas (such as limited drug supplies) filled. This fits with recent reports, such as the evaluation of the Free Health Care Initiative and the Partners In Health experiences of responding to the outbreak and ensuring future emergency preparedness [27, 28].

It is also important to re-establish not only services but strong links with the community, to regain their trust and involvement. The opportunity to ‘build back better’ health facility committees should be seized and the community health staff used more effectively to link communities and health facilities [20]. This is indeed anticipated in the post-Ebola plans including the Health Sector Recovery Plan 2015–2020 [6], the review of the HRH strategic plan 2015–2020 [29] and in the newly finalised Community Health Worker policy [30].

Building on health workers’ existing coping strategies is needed. Implementation research to better understand how peer networks and ICT can support health workers should be undertaken. In addition, the participants highlight the responsibility of the government to provide a safe health system, for both patients and staff. Three hundred seven health workers were infected with Ebola in Sierra Leone and 221 died (out of a reported total of 518 health worker deaths in the region during this epidemic) [31], and there is now a recognition that psychosocial support for them will need to be long-term [17, 32].

There are several limitations of this study. We were mindful that health workers were being asked to relive difficult experiences when the outbreak was still ongoing. For some health workers, this was the first opportunity to process these experiences, which proved to be distressing. The study was conducted as the outbreak was abating and we were conscious of not detracting from essential work by health workers and managers. Interviews were sometimes interrupted and cut short as respondents were needed elsewhere. This study draws on qualitative methods and explores the issues from the health workers’ and managers’ perspectives, which means that it cannot reveal other perspectives, such as those of the community and patients. The sample was limited as the aim was exploratory rather than to develop generalizable findings. The study did not include health workers from private facilities. They may have different experiences of the Ebola outbreak as well as other coping mechanisms, which need exploration.

## Conclusions

This study documents a very painful period with moving experiences of health workers as they continued to try to work and protect their households and communities. At the same time, it is clear that considerable reserves of health worker resilience were found. These patterns of resilience must be reinforced as the sector is rebuilt, both in Sierra Leone and elsewhere. Supportive supervision, peer support networks and better use of communication technology should be pursued, alongside a clear programme for rebuilding trust with community structures. Health workers are at the heart of the health system, and therefore listening to their voices about what helps them stay and do their job during a crisis is vital for building a responsive health system. The challenge is building these coping mechanisms into routine systems, pre-empting shocks, rather than waiting to respond belatedly to crises.

## Abbreviations

DHMT: District Health Management Team
EVD: Ebola Virus Disease
HRH: Human resources for health
HW: Health worker
IDI: In-depth interview
IPC: Infection prevention and control
KI: Key informant
KII: Key informant interview
PPE: Personal protective equipment
